# Proteomic Analyses of Chlorhexidine Tolerance Mechanisms in *Delftia acidovorans* Biofilms

**DOI:** 10.1128/mSphere.00017-15

**Published:** 2016-01-06

**Authors:** Tara Rema, Prabhakara Medihala, John R. Lawrence, Sinisa Vidovic, Gary G. Leppard, Marcia Reid, Darren R. Korber

**Affiliations:** a Food and Bioproduct Sciences, University of Saskatchewan, Saskatoon, Saskatchewan, Canada; b Environment Canada, Saskatoon, Saskatchewan, Canada; c Department of Veterinary and Biomedical Sciences, University of Minnesota, St. Paul, Minnesota, USA; d Environment Canada, Burlington, Ontario, Canada; e Electron Microscopy Facility, McMaster University, Hamilton, Ontario, Canada; AstraZeneca Pharmaceuticals

**Keywords:** *Delftia acidovorans*, *tolQ*, biofilms, chlorhexidine tolerance, protein expression

## Abstract

Protein expression and fatty acid profiles of biofilm cells of chlorhexidine-tolerant *Delftia acidovorans* (MIC = 15 µg/ml) and its chlorhexidine-susceptible mutant (MIC = 1 µg/ml) were investigated. The chlorhexidine-susceptible mutant (MT51) was derived from the parental strain (WT15) using Tn*5* transposon mutagenesis. The disrupted gene was identified as *tolQ*, a component of the *tolQRAB* gene cluster known to be involved in outer membrane stability. Proteomic responses of biofilm cells were compared by differential in-gel electrophoresis following exposure to chlorhexidine at sub-MIC (10 µg/ml) and above-MIC (30 µg/ml) concentrations. Numerous changes in protein abundance were observed in biofilm cells following chlorhexidine exposure, suggesting that molecular changes occurred during adaptation to chlorhexidine. Forty proteins showing significant differences (≥1.5-fold; *P* < 0.05) were identified by mass spectrometry and were associated with various functions, including amino acid and lipid biosynthesis, protein translation, energy metabolism, and stress-related functions (e.g., GroEL, aspartyl/glutamyl-tRNA amidotransferase, elongation factor Tu, Clp protease, and hydroxymyristoyl-ACP dehydratase). Several proteins involved in fatty acid synthesis were affected by chlorhexidine, in agreement with fatty acid analysis, wherein chlorhexidine-induced shifts in the fatty acid profile were observed in the chlorhexidine-tolerant cells, primarily the cyclic fatty acids. Transmission electron microscopy revealed more prominent changes in the cell envelope of chlorhexidine-susceptible MT51 cells. This study suggests that multiple mechanisms involving both the cell envelope (and likely TolQ) and panmetabolic regulation play roles in chlorhexidine tolerance in *D. acidovorans*.

**IMPORTANCE** *Delftia acidovorans* has been associated with a number of serious infections, including bacteremia, empyema, bacterial endocarditis, and ocular and urinary tract infections. It has also been linked with a variety of surface-associated nosocomial infections. Biofilm-forming antimicrobial-resistant *D. acidovorans* strains have also been isolated, including ones displaying resistance to the common broad-spectrum agent chlorhexidine. The mechanisms of chlorhexidine resistance in *D. acidovorans* are not known; hence, a chlorhexidine-susceptible mutant of the tolerant wild-type strain was obtained using transposon mutagenesis, and the proteome and ultrastructural changes of both strains were compared under chlorhexidine challenge.

## Abstract (precis)

*Delftia acidovorans* has been associated with a number of serious infections, including bacteremia, empyema, bacterial endocarditis, and ocular and urinary tract infections. It has also been linked with a variety of surface-associated nosocomial infections. Biofilm-forming antimicrobial-resistant *D. acidovorans* strains have also been isolated, including ones displaying resistance to the common broad-spectrum agent chlorhexidine. The mechanisms of chlorhexidine resistance in *D. acidovorans* are not known; hence, a chlorhexidine-susceptible mutant of the tolerant wild-type strain was obtained using transposon mutagenesis, and the proteome and ultrastructural changes of both strains were compared under chlorhexidine challenge.

## INTRODUCTION

Chlorhexidine is a broad-spectrum antimicrobial agent with wide application as an active ingredient in many daily-use disinfectants and pharmaceutical, personal-care, and health care products (1). Though chlorhexidine is often referred to by health professionals as a gold standard agent (2, 3) owing to its antibacterial efficacy, there are several reports of microorganisms surviving in the presence of in-use chlorhexidine concentrations (4–6). However, very little is known of the underlying mechanisms for this tolerance. Chlorhexidine tolerance levels are further elevated when bacteria live attached to surfaces as biofilms compared to their planktonic counterparts (7). Antimicrobial resistance mechanisms in biofilm bacteria have been well studied (7–9). However, there remains a dearth of understanding of the mechanisms involved in chlorhexidine resistance in microbial biofilms. The elucidation of the molecular details of antimicrobial resistance continues to be an active and valuable area of research. The few molecular studies conducted to date on chlorhexidine resistance have mainly relied upon genomic approaches (10–12). However, proteomic analysis has also proven valuable for antimicrobial resistance studies involving a variety of agents (13–16). An important distinction between genomic and proteomic approaches is that only expressed genes yielding functional proteins are detected in the proteome.

Gel-to-gel variability is a major drawback of gel-based techniques, leading to problems in detecting and quantifying differences in protein expression (17). Ünlü et al. (18) developed an approach involving the multiplexing of fluorescently labeled samples on the same gel; two-dimensional difference in-gel electrophoresis (DIGE). DIGE involves prelabeling of different protein samples with spectrally resolvable fluorescent dyes that are charge and mass matched, ensuring an equivalent impact on in-gel migration of labeled proteins and improving the overall accuracy of relative quantification between samples (19).

*Delftia acidovorans* is a Gram-negative bacterium ubiquitously found in soil and water and associated with a number of serious infections, including bacteremia, empyema, bacterial endocarditis, and ocular and urinary tract infections (20–22). *Delftia acidovorans* has also been implicated in various nosocomial biofilm infections associated with the use of medical devices like vascular catheters (23, 24), pressure-monitoring devices (25), and surgical instruments (26). Relatively little is known about *D. acidovorans*’ pathogenicity, prevalence, health and environmental risks, genetics, antibiotic resistance profile, and stress response mechanisms. Clinical *D. acidovorans* strains have shown resistance to β-lactams and related antibiotics, such as aminoglycosides and quinolones (27, 28). A strain resistant to chlorine was found in contaminated dental water units (29). More recently, *Delftia* spp. were shown to carry class 3 integrons, the genetic elements commonly associated with antibiotic resistance genes (30), indicating that this organism may horizontally acquire resistance genes from microbes in the environment.

A chlorhexidine-tolerant *D. acidovorans* strain (WT15) (MIC = 15 µg/ml) was isolated from a river biofilm and characterized (31). A chlorhexidine-susceptible mutant (MT51) (MIC = 1 µg/ml) was derived from WT15 using Tn*5* transposon mutagenesis (31). We then used whole-proteome analysis of chlorhexidine-tolerant and chlorhexidine-susceptible *D. acidovorans* biofilms grown in the presence and absence of chlorhexidine in order to examine the adaptive response.

## RESULTS

### Identification of transposon insertion site and antimicrobial resistance patterns.

To elucidate the molecular mechanism of chlorhexidine tolerance in *D. acidovorans*, we carried out transposon mutagenesis (31) to create a chlorhexidine-susceptible mutant strain (MT51). The MIC of strain MT51 (1 µg/ml) for chlorhexidine was fifteen times lower than that of the parent strain WT15 (15 µg/ml). Identification of the DNA sequence flanking the transposon insertion site in this mutant revealed that the transposon was inserted into the 81st base pair of the 705-bp DNA region encoding the protein, TolQ. The sequence of this gene was 100% identical to the *D. acidovorans* strain SPH1 *tolQ* sequence published in the Nucleotide Database of the National Center for Biotechnology Information (NCBI). Apart from chlorhexidine susceptibility, transposon mutation also rendered the MT51 strain less resistant to amikacin (MICs of 16 and 64 µg/ml for MT51 and WT15, respectively) among the 17 antibiotics in the National Antimicrobial Resistance Monitoring System of the Centers for Disease Control and Prevention panel (Table 1). MICs for the other 16 antimicrobial agents in the screened panel were not observed to change appreciably following mutagenesis.

**TABLE 1:** Results of antibiotic and chlorhexidine susceptibility testing for *D. acidovorans* strains WT51 (wild type) and MT51 (mutant)

Antibiotic	MIC (µg/ml)	
	Wild type	Mutant
Amikacin^a^	64	16
Chloramphenicol	16	16
Cefoxitin	<0.5	<0.5
Tetracycline	<4.0	<4.0
Ceftriaxone	<0.25	0.5
Amoxicillin	1	1
Clavulanic acid	0.5	0.5
Ciprofloxacin	0.25	0.12
Gentamicin	16	16
Nalidixic acid	0.5	0.5
Ceftiofur	0.5	0.5
Sulfisoxazole	32	32
Trimethoprim	0.12	0.12
Sulfamethoxazole	2.38	2.38
Kanamycin	64	64
Ampicillin	32	32
Streptomycin	64	64
Chlorhexidine^a^	15	1

^a^ Compound for which a difference in MIC was observed.

### TEM.

**FIG 1:**
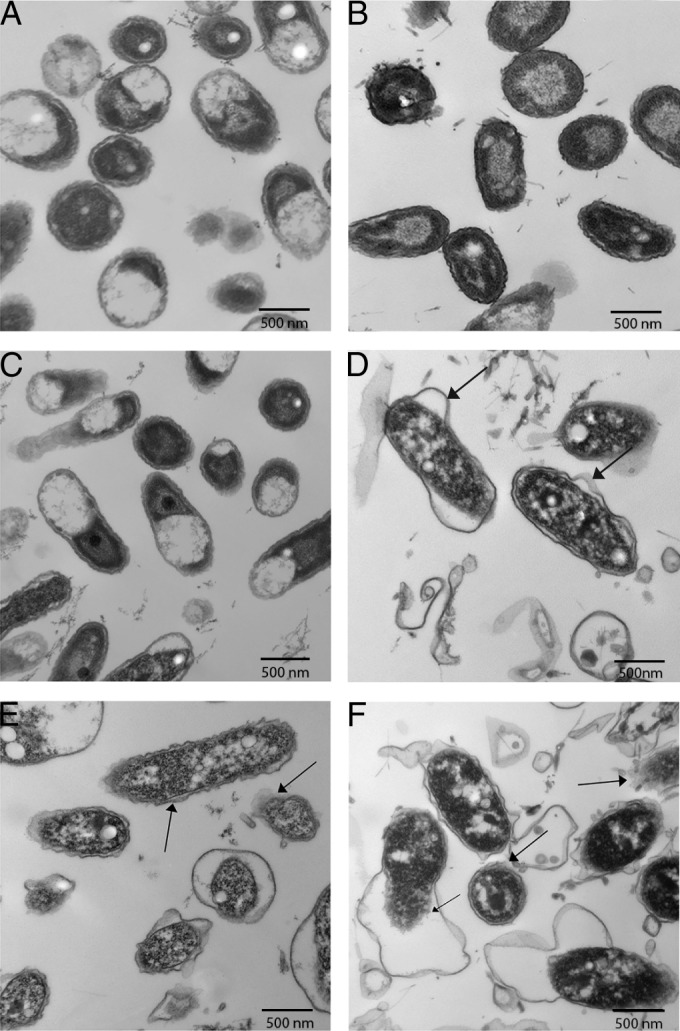
TEM micrographs of 48 h *D. acidovorans* biofilm cells with and without exposure to 10 and 30 µg/ml chlorhexidine after 24 h of growth. (A) WT15 control; (B) MT51 control; (C) WT15 treated with chlorhexidine at 10 µg/ml; (D) MT51 treated with chlorhexidine at 10 µg/ml; (E) WT15 treated with chlorhexidine at 30 µg/ml; (F) MT51 treated with chlorhexidine at 30 µg/ml. Arrowheads show membrane structural alterations. Bar, 500 nm.

Transmission electron microscopy (TEM) was used to study the ultrastructural effects of chlorhexidine in both chlorhexidine-tolerant and chlorhexidine-susceptible *D. acidovorans* biofilm cells. Figure 1A and B show electron micrographs of cells recovered from 48-h untreated control WT15 and MT51 biofilms, respectively, and thus lack apparent damage to the cell envelope or coagulation of the cytoplasm. The WT15 biofilm cells treated at 10 µg/ml (Fig. 1C) appear similar to the WT15 untreated cells; however, the 10-µg/ml chlorhexidine treatment produced an effect on MT51, especially at the cell envelope, where membrane bulging and detachment of cell membrane from the cell wall were observed (see arrows in Fig. 1D). Similar membrane waviness and damage were apparent in both MT51 and WT15 biofilms treated with 30 µg/ml chlorhexidine (Fig. 1E and F), suggesting that a lethal dose of chlorhexidine exerts membrane-level effects on *D. acidovorans* cells.

### Cellular fatty acid analysis.

**FIG 2:**
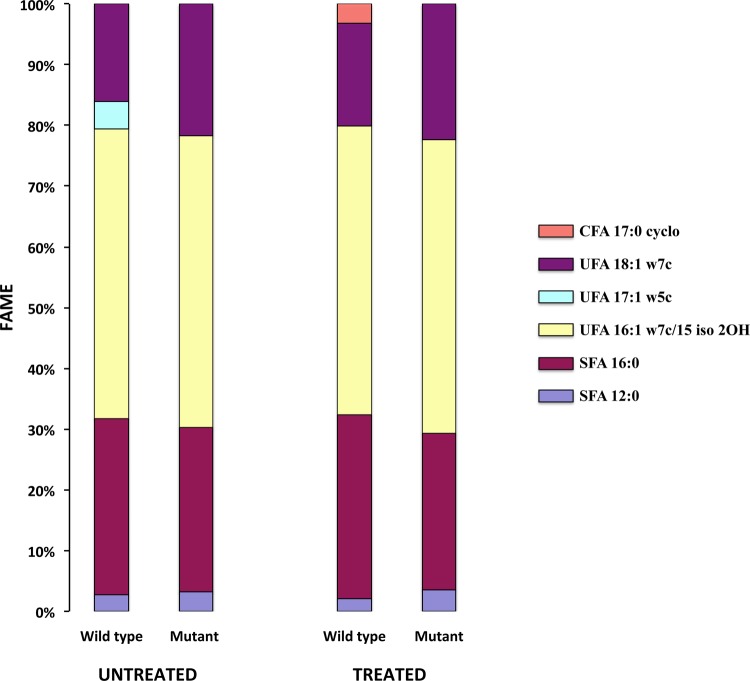
Relative amounts of fatty acid methyl esters, determined using gas chromatography, found in chlorhexidine-tolerant (WT15) and chlorhexidine-susceptible (MT51) *D. acidovorans* biofilm cells treated with (10 µg/ml with chlorhexidine) compared with the untreated control. CFA, cyclo fatty acids; UFA, unsaturated fatty acids; SFA, saturated fatty acids.

Treated (10 µg/ml chlorhexidine) and untreated biofilms of chlorhexidine-tolerant WT15 and chlorhexidine-susceptible MT51 biofilms were subjected to fatty acid methyl ester (FAME) analysis. The fatty acids detected in WT15 control biofilms after 48 h of growth (Fig. 2) included both saturated fatty acids (12:0 and 16:0) and unsaturated fatty acids (16:1ω7c/15 iso2OH, 17:1ω5c, and 18:1ω7c). Treatment of chlorhexidine-tolerant WT15 biofilms with 10 µg/ml chlorhexidine resulted in a reduction in the concentration of total unsaturated fatty acids from an initial value of 68.2% to 63.6% (Fig. 2), apparently due to the disappearance of 17:1ω5c fatty acid. Simultaneously, there was a minor increase in saturated fatty acids and the appearance of a new group, the cyclic fatty acids (17:0 cyclo), in the chlorhexidine-treated wild-type biofilms. The unsaturated fatty acid, 17:1ω5c, was not found in mutant MT51 biofilms. When MT51 biofilms were exposed to chlorhexidine (10 µg/ml), the level of unsaturated fatty acids increased slightly from 68.4% to 70.1%, and no production of cyclic fatty acids was observed.

### Proteomic analyses.

**FIG 3:**
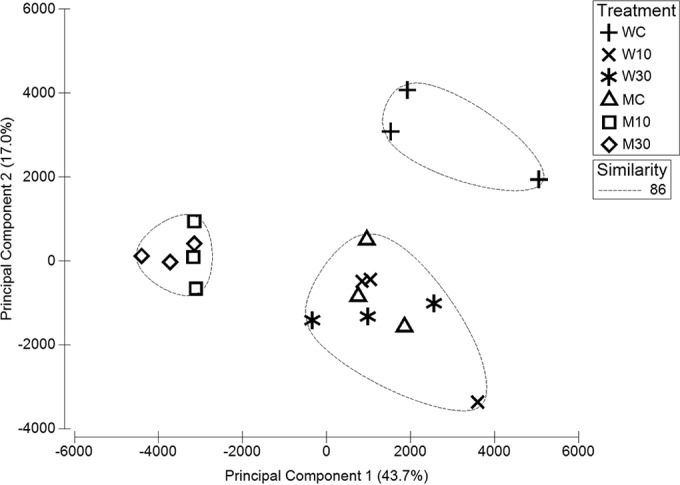
Principal component analysis of the 18 individual DIGE expression spot maps (6 treatments with three replications per treatment) differentiated by principal components one and two. Statistical analyses of PCA scores generated from the first two component axes were run using an analysis of similarity (ANOSIM) with PRIMER v6 software. M10 and M30 are the mutant strain treated with 10 and 30 µg/ml chlorhexidine, respectively; W10 and W30 are the wild-type strain treated with 10 and 30 µg/ml chlorhexidine, respectively; MC and WC are the mutant and wild-type controls, respectively.

DIGE analyses were carried out using (i) 48-h WT15 and MT51 control biofilms receiving no chlorhexidine and (ii) WT15 and MT51 biofilms treated for 24 h with 10 and 30 µg/ml chlorhexidine after 24 h of initial biofilm growth. Principal component analysis (PCA) was used to group the 18 individual Cy3- or Cy5-labeled spot maps based on the overall expression pattern of all 999 spots matched between gels and to identify any experimental outliers. The PCA of spot maps (Fig. 3) demonstrated a high reproducibility between replicate samples, as indicated by close grouping of the experimental replicates. The first principal component accounted for 43.7% of total variance and clearly separated the MT51 control, MT51 treated (10 µg/ml), and WT15 control groups from each other and indicated little separation between the WT15 control and WT15 treated biofilms. However, the second principal component, which accounted for 17% of the variance, differentiated WT15 treated groups from the other experimental groups. PCA results indicated significant differences between mutant and wild-type control biofilms (analysis of similarity [ANOSIM], *P* < 0.001). Mutant treated biofilms tended to group together (ANOSIM, *P* < 0.001), indicating similar proteomic responses. Overall, PCA demonstrated that both strains were differentially affected by chlorhexidine treatment relative to their untreated controls.

**FIG 4:**
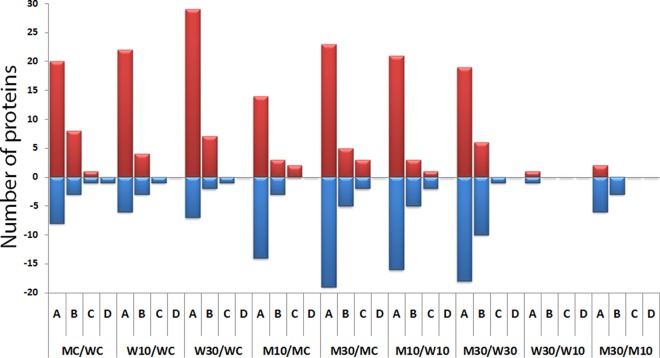
Number of up-regulated and down-regulated proteins among various levels of significant change between various experimental groups. Red bars, up-regulated proteins; blue bars, down-regulated proteins. A, B, C, and D represent 2- to 3-fold, 3- to 5-fold, 5- to 10-fold, and >10-fold changes, respectively (*P* < 0.05). M10 and M30 are the mutant strain treated with 10 and 30 µg/ml chlorhexidine, respectively; W10 and W30 are the wild-type strain treated with 10 and 30 µg/ml chlorhexidine, respectively; MC and WC are the mutant and wild-type controls, respectively.

DIGE analysis revealed numerous protein species abundance changes in both wild-type and mutant biofilms when exposed to sub-MIC (10 µg/ml) and above-MIC (30 µg/ml) chlorhexidine levels. The number of differentially expressed proteins among the various groups compared are shown in Table 2. For example, 114 proteins were differentially expressed between untreated control WT15 and MT51 biofilms, of which 70 were up-regulated and 44 were down-regulated in MT51. Table 2 also shows that the number of differentially expressed proteins with respect to the two chlorhexidine concentrations was 9 (5 were up-regulated and 4 were down-regulated) in WT15 biofilms, whereas 56 proteins (30 were up-regulated and 26 were down-regulated) were differentially expressed in MT51 biofilms. This finding is in keeping with our TEM data (Fig. 1), which showed that MT51 was more dramatically affected by chlorhexidine treatment. Two-way analysis of variance (ANOVA) showed that treatment-specific differential expression of proteins was more profound than the effect of the *tolQ* mutation (*P* < 0.05), as 106 proteins were differentially expressed (among the mutant and wild type) across all treatments, whereas only 60 proteins were affected due to transposon insertion. A further breakdown of the differentially expressed proteins, according to the degree of change, is shown in Fig. 4. Chlorhexidine treatment induced higher changes (>1.5-fold) in protein expression in both WT15 and MT51 biofilms than those between the respective untreated controls. However, the protein expression changes in the same biofilm treated with 30 µg/ml chlorhexidine were higher in MT51 than the WT15 biofilms relative to their respective biofilms exposed to 10 µg/ml. Only one spot each showed increased and decreased abundance of 2- to 3-fold in wild-type biofilms treated with high versus low concentrations of chlorhexidine, whereas 2 spots with increases and 7 spots with decreases in abundances, 2- to 3-fold, were seen in mutant biofilms.

**TABLE 2:** Comparison data of differentially expressed proteins (≥1.5-fold increase/decrease; *P* < 0.05) among various experimental groups^a^

Groups compared for analysis	No. of proteins				Greatest fold change	
	Differentially expressed	Picked for identification	With increased abundance	With decreased abundance	Increase	Decrease
MC and WC	114	25	70	44	6.28	15.19
W10 and WC	105	26	53	52	6.25	7.54
W30 and WC	83	27	49	34	14.87	7.49
M10 and MC	107	30	63	44	7.62	4.47
M30 and MC	119	39	69	50	7.0	7.14
M10 and W10	138	40	92	46	5.4	5.75
M30 and W30	111	30	39	72	4.43	8.26
W30 and W10	9	4	5	4	2.36	2.77
M30 and M10	56	11	30	26	2.25	3.62
2-way ANOVA (strain)	60	13	NA	NA	NA	NA
2-way ANOVA (treatment)	106	31	NA	NA	NA	NA
2-way ANOVA (interaction)	54	14	NA	NA	NA	NA

^a^ NA, not applicable.

**FIG 5:**
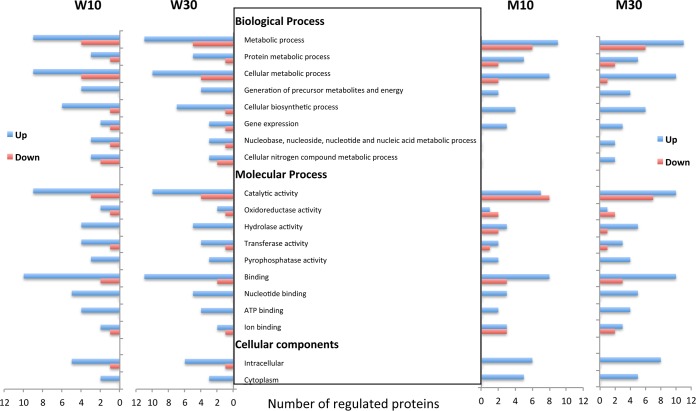
Gene ontology cluster (DAVID) analysis of proteins up-regulated (blue) and down-regulated (red) in WT15 and MT51 biofilms treated with chlorhexidine at 10 and 30 µg/ml showing the biological, molecular, and cellular processes that were affected. The proteins were picked from the Deep Purple-stained pick gel and analyzed by mass spectrometry.

Out of 45 differentially expressed proteins picked, 40 proteins were successfully identified and are summarized in Table 3, along with their molecular functions, accession numbers, and changes in abundance. Based on their known functions and Database for Annotation, Visualization and Integrated Discovery (DAVID) analysis, these identified proteins were broadly categorized as those involved in amino acid and lipid biosynthesis, translation/transcription, energy metabolism, and membrane and stress-related functions. DAVID analysis (Fig. 5) shows the major biological and molecular processes that were affected due to chlorhexidine treatment in WT15 and MT51 biofilms. The most pronounced changes observed were the down-regulation of proteins involved in biological processes, as listed in Fig. 5. Of the 40 identified proteins, 12 proteins were up-regulated in both strains (WT15 and MT51) at both concentrations of chlorhexidine. These included chaperonin GroEL, aspartyl/glutamyl-tRNA amidotransferase subunit A, F_o_F_1_ ATP synthase subunit alpha, elongation factors Tu and Ts, phosphopyruvate hydratase, amidohydrolase, basic membrane lipoprotein, electron transfer flavoprotein, and ATP-dependent Clp protease. Those that were down-regulated in both WT15 and MT51 biofilms included ribosomal subunit interface protein, AMP-binding domain-containing protein, and a hypothetical protein (ferritin-like superfamily). The chaperonin protein ClpP (spot no. 658) (Table 3) was found to be up-regulated in WT15 biofilms exposed to the inhibitory (30 µg/ml) chlorhexidine concentration, and its levels were higher and almost similar in both 10-µg/ml- and 30-µg/ml-treated MT51 biofilms, suggesting that both these chlorhexidine concentrations were stressful to MT51. Similarly, the chaperonin GroEL was also significantly up-regulated in MT51 chlorhexidine-treated biofilms compared with WT15 chlorhexidine-treated biofilms. Several protein spots with slightly different electrophoretic migration patterns were determined to be the same protein (i.e., chaperonin GroEL, ATP synthase, and elongation factor Tu), which may imply that charge or posttranslational modifications had occurred (32). Another protein, the small heat shock protein HSP 20 (spot no. 886) (Table 3), which is an ATP-independent molecular chaperone, was also significantly down-regulated nearly 7-fold only in wild-type chlorhexidine-treated biofilms. There was also a >2-fold decrease in expression of phasin proteins (Table 3) in WT15 chlorhexidine-exposed biofilms compared to MT51 biofilms. Oxidative stress proteins, namely, superoxide dismutase, a hypothetical protein (alkylhydroperoxidase-like protein, AhpD family), and redoxin domain-containing protein, either were down-regulated or showed no significant changes in WT15 and MT51 biofilms (Table 3).

**TABLE 3:** Protein expression changes in 48 h *D. acidovorans* WT15 and MT51 biofilms following chlorhexidine treatment at 0, 10 (W10 and M10), and 30 (W30 and M30) µg/ml after 24 h of biofilm growth

Biological process and spot no.**	Protein	Accession no.	Mass	pI	Fold change^b^							2-way ANOVA^c^
					MC/WC	W10	W30	M10	M30	M10/W10	M30/W30	
Generation of precursor metabolites and energy (GO:0006091)^a^												
115	ATP synthase subunit alpha	gi|160895867	55,205	5.64	+4.07	+2.76	+2.91	NS	NS	NS	+2.16	S, T, I
122	ATP synthase subunit alpha	gi|160895867	55,205	5.64	+2.81	+1.90	+2.45	NS	+1.81	+2.07	+2.07	S, T, I
125	ATP synthase subunit beta	gi|160895869	50,633	5.23	+2.68	+2.22	+3.08	NS	+2.84	+1.98	+2.48	S, T, I
995	ATP synthase subunit beta	gi|160895869	50,633	5.23	+2.01	+2.12	+2.24	+1.88	+1.75	+1.79	NS	S, T
589	Ubiquinol-cytochrome *c* reductase iron-sulfur subunit	gi|160900935	21,695	6.1	NS	−1.84	−2.80	NS	NS	NS	NS	T, I
200	Enolase	gi|160900540	45,951	4.82	+2.24	+2.92	+3.27	+2.98	+3.54	+2.29	+2.43	S, T
Fatty acid metabolic process (GO:0006631)												
478	Malonyl CoA-acyl carrier protein transacylase	gi|160900703	34,368	5.32	+2.21	+1.35	+1.82	NS	NS	NS	NS	S, T
770	(3*R*)-Hydroxymyristoyl-ACP dehydratase	gi|160900369	16,579	5.92	NS	NS	NS	+1.72	+2.22	NS	NS	S, T, I
563	Enoyl-CoA hydratase/isomerase	gi|160896976	27,994	5.39	+2.50	NS	NS	−2.63	−3.82	NS	NS	T, I
371	Basic membrane lipoprotein	gi|160897585	41,068	5.71	NS	+3.30	+6.33	+7.62	+6.20	NS	NS	S, T, I
Nucleotide biosynthetic process (GO:0009165)												
606	Glutamine amidotransferase	gi|160899150	25,022	5.72	−15.19	−3.63	−3.42	NS	NS	−5.09	−3.46	S, T, I
218	Amidohydrolase	gi|160897029	45,940	5.03	NS	+2.11	+2.43	+1.92	+2.16	NS	NS	S, T
91	Bifunctional phosphoribosylaminoimidazole-carboxamide formyltransferase/IMP cyclohydrolase	gi|160897011	57,189	5.72	+2.26	+1.79	+2.18	NS	NS	NS	NS	S, T
Molecular chaperones / protein folding (GO:0006457)												
877	FKBP-type peptidylprolyl isomerase	gi|160900492	12,314	5.23	−2.02	−1.73	−1.53	NS	NS	−1.59	−1.83	S, T
544	PpiC-type peptidyl-prolyl *cis*-*trans* isomerase	gi|160898739	28,914	8.5	−1.69	NS	NS	−1.50	−2.33	−1.93	−2.58	S, T
886	Heat shock protein HSP20	gi|160897822	13,608	5.78	−9.43	−7.54	−7.49	NS	NS			S, T, I
59	60-kDa chaperonin	gi|160901092	57,123	5.02	NS	+1.85	+3.36	+3.35	+ 7.0	+1.59	+1.84	S, T, I
85	60-kDa chaperonin	gi|160901092	57,123	5.02	NS	NS	NS	NS	+2.92	+2.05	+2.14	S, T, I
86	60-kDa chaperonin	gi|160901092	57,123	5.02	NS	NS	NS	+2.03	+2.49	+1.77	+1.89	S, T, I
Translation/transcription (GO:0006412/0006351)												
95	Aspartyl/glutamyl-tRNA amidotransferase subunit A	gi|160895801	52,727	5.51	+2.30	+2.47	+2.65	+2.19	+2.35	+2.04	NS	S, T
190	Elongation factor Tu	gi|160895838	43,296	5.48	NS	+2.06	+2.38	+2.53	+3.25	NS	+2.42	S, T, I
274	Elongation factor Tu	gi|160895838	43,296	5.48	NS	+3.81	+4.31	NS	+2.01	NS	NS	T, I
436	Elongation factor Ts	gi|160900379	31,142	5.5	NS	NS	+2.07	+1.76	+2.06	NS	NS	T
629	Two-component LuxR family transcriptional regulator	gi|160899416	23,896	5.87	NS	−1.64	−1.97	NS	NS	NS	NS	S, T, I
Electron carrier activity (GO:0009055)												
292	Taurine catabolism dioxygenase TauD/TfdA	gi|160900189	37,395	5.17	NS	+2.17	+2.49	NS	NS	−1.83	NS	S, T, I
612	Electron transfer flavoprotein subunit alpha/beta	gi|160896849	26,734	7.64	NS	NS	+1.66	+1.96	+3.08	+1.57	+1.67	
Oxygen and reactive oxygen species metabolic process (GO:0006800)												
662	Superoxide dismutase	gi|160899011	21,614	5.87	NS	−3.76	−4.56	NS	NS	NS	+3.39	S, T, I
647	Superoxide dismutase	gi|16089811	22797	5.86	−2.19	NS	NS	NS	NS	NS	−3.62	
644	Hypothetical protein (alkylhydroperoxidase-like protein, AhpD family)	gi|160899719	23,967	5.87	NS	NS	NS	−3.03	NS	−2.92	NS	S, T, I
					
Proteolysis (GO:0006508)												
658	ATP-dependent Clp protease proteolytic subunit	gi|160898086	22,274	5.45	NS	NS	+1.49	+1.77	+1.87	NS	NS	S, T, I
75	Hypothetical protein (peptidase dimerization domain protein)	gi|160896656	62,743	7.08	NS	NS	NS	−2.66	−2.48	−2.58	−2.51	S, T, I
Antibiotic metabolic process (GO:0016999)												
536	Hydroxyacylglutathione hydrolase	gi|160900329	28,066	5.34	+6.28	NS	NS	−2.59	NS	+3.77	+4.23	S, T
					
Phosphorus metabolic process (GO:0006793)												
687	Inorganic diphosphatase	gi|160900311	19,368	4.96	NS	NS	NS	+1.43	+2.02	NS	NS	T, I
					
Cell redox homeostasis												
734	Redoxin domain-containing protein	gi|160895850	20,383	5.63	NS	NS	NS	NS	−2.78	NS	NS	S, T, I
					
Transaminase activity (GO:0008483)												
319	Class V aminotransferase	gi|160896522	41,574	6.08	NS	NS	NS	−1.96	−6.12	−2.05	−4.33	S, T, I
					
Ion bonding (GO:0043167)												
416	Hypothetical protein (ferritin-like superfamily of di-iron-containing four-helix-bundle protein)	gi|160901296	34,343	4.86	+3.97	NS	NS	−2.45	−2.71	+2.23	+2.26	S, T
					
Others/unknown												
595	Glutathione *S*-transferase domain-containing protein	gi|160896300	23,088	6.17	NS	NS	NS	NS	+2.41	+1.73	NS	S, T, I
808	Ribosomal subunit interface protein	gi|160896367	13,610	5.51	−1.96	NS	NS	NS	−1.96	−1.87	−2.64	S, T
46	AMP-binding domain-containing protein	gi|160901406	63,817	5.63	NS	NS	−1.62	−4.47	−7.14	−3.13	−2.98	S, T, I
714	Phasin family protein	gi|160900174	19,110	5.43	−2.35	−2.14	−2.11	NS	NS	NS	NS	S, T, I

^a^ GO, gene ontology ID.

^b^ Fold change in protein expression from the respective control biofilms. Minus and plus signs indicate decreased and increased expression. NS, not significant.

^c^ S, strain (WT15 and MT51); T, treatment (0, 10, and 30 μg ml−1); I, interaction.

Consistent with our FAME results, several enzymes associated with fatty acid synthesis were also found to be affected by chlorhexidine treatment in both WT15 and MT51 biofilms. They included malonyl coenzyme A (CoA)-acyl carrier protein transacylase (spot no. 478), enoyl-CoA hydratase/isomerase (spot no. 563), and (3R)-hydroxymyristoyl-ACP dehydratase (spot no. 770). A dramatic increase, from ~3.3- to 7.5-fold, in the expression of a basic membrane lipoprotein (spot no. 371) (Table 3) in both WT15 and MT51 chlorhexidine-treated biofilms indicated that this protein may play a very important role in chlorhexidine tolerance in *D. acidovorans*. A role for quorum sensing is also suggested by the down-regulation of the two-component LuxR family transcriptional regulator (spot no. 629) (Table 3), especially in WT15 biofilms treated with the high concentration of chlorhexidine.

Chlorhexidine treatment also doubled the abundance of amidohydrolase enzyme in both WT15 and MT51 biofilms (spot no. 218) (Table 3). Similarly, proteins involved in protein synthesis, folding, and stabilization (GroEL, ClpP protease, aspartyl/glutamyl-tRNA amidotransferase, and elongation factors Tu and Ts) and a few proteins associated with energy production, nucleotide transport, and metabolism were also up-regulated (i.e., bifunctional phosphoribosylaminoimidazolecarboxamide formyltransferase/IMP cyclohydrolase, phosphopyruvate hydratase, F_o_F_1_ ATP synthase subunit alpha/beta, and electron transfer flavoprotein). However, the enzyme glutamine amidotransferase (spot no. 606) (Table 3), also involved in nucleotide transport and metabolism, was down-regulated by a factor of almost 3.5 in WT15 biofilms at both chlorhexidine concentrations, whereas the taurine catabolism dioxygenase TauD/TfdA (spot no. 292) was up-regulated more than 2-fold in WT15 biofilms treated with chlorhexidine.

The major differences between the wild-type and mutant control biofilms were the up-regulation of the ATP synthases, a few enzymes involved in fatty acid metabolism, and hydroxyacylglutathione hydrolase and the down-regulation of glutamine amidotransferase, several molecular chaperones, and family phasin protein in mutant biofilms compared to their parent biofilms (Table 3).

## DISCUSSION

Mutation of *tol* genes has been shown to confer hypersensitivity to various agents, such as detergents, quaternary compounds, and some antibiotics, in *Escherichia coli* (33). However, little is known about TolQ’s functional role in *D. acidovorans*. In *E. coli*, TolQ is an integral cytoplasmic membrane protein (230 amino acids) with three membrane-spanning regions required for maintenance of the integrity of the bacterial envelope (34). The *tolQRAB-pal* operon also functions in the uptake of certain bacteriophages and colicins and is conserved in Gram-negative genomes (33). Studies also suggest that the Tol/Pal system might anchor the OM to the peptidoglycan layer (35) and further may catalyze porin biogenesis or activity (36). It was thus hypothesized that a mutation in *tolQ* in *D. acidovorans* would affect the cell envelope’s integrity upon exposure to chlorhexidine, a membrane-active agent (37). Due to the absence of complete knowledge of TolQ in *D. acidovorans*, along with limitations in the present study, it is possible that TolQ influenced the activity of other proteins not detected in our DIGE analysis. For example, Omp32, undetected in our analyses, has been found to have strong anion selectivity and can repulse the penetration of positively charged compounds such as chlorhexidine (38). Previously, we demonstrated that more chlorhexidine accumulated inside chlorhexidine-susceptible MT51 cells than in chlorhexidine-tolerant cells (31), clearly pointing to selectivity in chlorhexidine penetration.

TEM has been used extensively to study the effects of antimicrobial agents on microorganisms (39, 40). Our TEM analyses provided clear evidence that chlorhexidine has a disruptive effect on the *D. acidovorans* cell envelope, in agreement with previous reports for other bacteria (41–43). These observations are also consistent with the *tolQ* mutation influencing membrane integrity of *D. acidovorans* cells, where the mutant cells appeared more susceptible to chlorhexidine than the wild type (Fig. 1). Tattawasart et al. (44) observed that chlorhexidine-resistant *Pseudomonas stutzeri* cells were larger and had fibril-like structures on their outer surfaces and that the cell envelope was wavier and thicker than those of susceptible isolates. Time-dependent cellular damage (i.e., blebbing of the outer membrane and cytoplasmic swelling) was also seen in the case of susceptible isolates treated with chlorhexidine (44). These authors also observed extensive cell lysis, likely the consequence of exposing the cells to a significantly higher chlorhexidine concentration (100 µg/ml) than that employed in our study. The type of bacteria (planktonic versus biofilm), chlorhexidine concentration, and period of exposure all play important roles in inducing ultrastructural changes in microorganisms (40).

Changes in cell fatty acid composition in the presence of chlorhexidine possibly reflect alterations in membrane fluidity and symmetry and offer a potential mechanism of chlorhexidine tolerance in *D. acidovorans*. There were some differences in the major fatty acids detected in our chlorhexidine-tolerant *D. acidovorans* strain (Fig. 2) after a 48-h treatment compared with the *D. acidovorans* strain of Wen et al. (45). These differences were likely due to the combined effect of using biofilms (our study) versus planktonic cells, as well as differences in growth conditions. In our study, chlorhexidine treatment caused a reduction in total unsaturated fatty acids associated with the disappearance of the 17:1ω5c fatty acid and the *de novo* appearance of 17:0 cyclo fatty acids in the wild type strain. Such a change in membrane fatty acid composition has been associated with decreased membrane fluidity (46, 47). Abu-Elteen and Whittaker (48) observed that the percentage of unsaturated fatty acids decreased while the ratios of C16:C18 increased in the presence of chlorhexidine in *Candida albicans*. Chlorhexidine has also been observed to alter the cell membrane lipid packing order and membrane fluidity in human epithelial cells (49). The fatty acid compositional modifications (Fig. 2) that were seen in WT15 biofilms in response to chlorhexidine treatment were not evident in the mutant chlorhexidine-treated biofilm and thus appear to be part of the *D. acidovorans* adaptive response.

DIGE has previously been used to elucidate the proteomes of bacterial drug resistance (32, 50, 51). Our study is the first to investigate chlorhexidine-induced changes in the *D. acidovorans* proteome. PCA analysis of the DIGE gels (Fig. 3) indicated that the effect of chlorhexidine on MT51 biofilm protein expression was distinct from its effect on the WT15 biofilms and that both chlorhexidine-treated biofilms were different from their respective control biofilm conditions. These differences appear due to the mutation in the *tolQ* gene in MT51, as TolQ has previously been shown to influence antimicrobial resistance and membrane integrity in other bacterial cells (33, 34). PCA analyses also confirmed significant differences (ANOSIM, *P* < 0.001) between WT15 and MT51 control biofilms due to *tolQ* mutation. Since WT15 is chlorhexidine tolerant (31) (Table 1), we did not expect a large difference between the control and chlorhexidine-treated proteomes, especially at the lower concentration. In contrast, both low and high chlorhexidine concentrations used in this study were above the MIC determined for MT51 cells, and so MT51 treated biofilms were significantly different from MT51 control biofilms (ANOSIM, *P* < 0.001). Hence, it was hypothesized that the MT51 biofilms would experience greater chlorhexidine-induced stress than the WT15 biofilms, and this would be reflected by their proteome response. Previously, microscopic analyses (31) also showed greater effects of chlorhexidine on MT51 biofilms. Accordingly, the first principal component (PC1) showed that a greater differential effect on protein expression did in fact result from application of the two chlorhexidine concentrations (10 and 30 µg/ml) on MT51 biofilms.

Bacterial stress response is a coordinated outcome of the expression of a variety of genes that alter various cellular processes (cell division, DNA metabolism, housekeeping, membrane composition, transport, etc.) and the genes involved are numerous (52). Information on the proteomes of bacterial species exposed to chlorhexidine, or on those of chlorhexidine-resistant isolates, is limited. Recently, Coenye et al. (12) used microarray analysis to assess molecular mechanisms of chlorhexidine tolerance in *Burkholderia cenocepacia* biofilms, revealing that a 15-min exposure of *B. cenocepacia* to 0.15 µg/ml chlorhexidine resulted in the up-regulation of 469 (6.56% of the total) and down-regulation of 257 (3.59% of the total) protein-coding genes (>2-fold change; *P* < 0.05). Similarly, the analysis of DIGE gels in our study revealed numerous protein species abundance changes in both wild-type and mutant biofilms that had been exposed to sub- and above-MIC levels of chlorhexidine (Table 2). A greater number of proteins were affected (Fig. 4) in chlorhexidine-treated versus untreated mutant biofilms than in the treated versus untreated wild-type biofilms, suggesting that the MT51 mutant was more dramatically affected by chlorhexidine exposure, in keeping with our TEM and other observations. Two-way ANOVA (*P* < 0.05) analysis (Table 3) showed that treatment-specific differential expression was profound compared to that based on strain, as the proteome response in MT51 cells was the integrated effect of the mutated genetic background as well as above-MIC chlorhexidine exposure.

A wide range of key enzymes and proteins involved in various biological and molecular processes were affected by chlorhexidine, as shown by gene ontology analysis (Fig. 5). The DIGE proteomic data revealed that the differentially expressed proteins in these various treatment groups fell into several functional groups, mainly those involved in energy, nucleotide, fatty acid, and amino acid metabolism, protein translation and modification, DNA binding and transcription, cell membrane-related functions, and other cellular processes, such as detoxification and stress response (Table 3). The cytoplasmic membrane would be the highest-probability target for biocidal action for chlorhexidine, causing functional perturbation of the membrane (53). This implies that the target enzymes and proteins for biocide action would be mostly those related to structural integrity, transport mechanisms, energy-coupling processes, and respiratory chain function and those that are membrane bound (53), and this was consistent with our observations (Table 3). The majority of *B. cenocepacia* biofilm protein-coding genes overexpressed when cells were treated with chlorhexidine are those coding for periplasmic and exporter proteins or lipoproteins and efflux systems and those that are required for structure and function of the inner membrane, transport or binding, and chemotaxis and motility (12). The majority of down-regulated protein-coding genes were involved in transport or binding or regulatory functions. Similarly, treatment of *Pseudomonas aeruginosa* planktonic cells with 0.008 mM chlorhexidine for 10 and 60 min revealed that membrane transport, oxidative phosphorylation, and electron transport genes were down-regulated (54). The *oprH* gene and MexCD-OprJ multidrug efflux pump were among the up-regulated proteins found to play a major role in chlorhexidine resistance in *P. aeruginosa* (54).

In our study, several stress response-related proteins were found to be differentially expressed in the presence of chlorhexidine, including GroEL and ATP synthase. Other authors have made similar observations in organisms under antimicrobial stress (51, 55, 56). Overexpression of GroEL in MT51 biofilms may be directly related to the sensitivity of this strain to chlorhexidine and their stressed metabolic state. It further indicates that there may be an increased concentration of misfolded proteins in MT51 biofilms and chlorhexidine may interfere with protein synthesis or structure (55). Similarly, there is evidence for the role of housekeeping genes, such as glutamyl-tRNA amidotransferase and elongation factors Tu and Ts, in biocide tolerance (39). Clp protease is a periplasmic chaperone protein that plays a major role in bacterial survival under stress conditions where proteins tend to unfold and aggregate (32, 57). ClpP was found to be up-regulated in both WT15 and MT51 upon chlorhexidine treatment, though the induction level of ClpP was higher at both concentrations in MT15 biofilms. In *Staphylococcus aureus*, Clp ATPases are required for stress tolerance against a broad range of inducing factors (57). Induction of this protease upon chlorhexidine treatment indicates an effect on periplasmic proteins in order to decrease the toxic effect of misfolded proteins. However, the small heat shock protein, HSP 20, which is an ATP-independent molecular chaperone, was down-regulated nearly 7-fold in WT15 chlorhexidine-treated biofilms, indicating that this protein did not play a significant role in chlorhexidine tolerance (58). Superoxide dismutase was also down-regulated in WT15 biofilms and not changed significantly in mutant biofilms, suggesting the absence of oxidative stress during chlorhexidine exposure. Other studies have made similar findings using different antimicrobial agents (32). The lack of oxidative stress is further substantiated by the down-regulation or absence of change in the hypothetical protein (alkylhydroperoxidase-like protein, AhpD family) and redoxin domain-containing protein, which may have a role in attenuating the oxidative stress caused by peroxides and other reactive oxygen species (59). Seemingly, the down-regulation of these proteins may not be necessary to combat chlorhexidine stress, which may reflect the balanced utilization of useful proteins to overcome chlorhexidine stress.

The DIGE data also suggest the possibility of detoxification or degradation of chlorhexidine by amidohydrolases. The amidohydrolase superfamily is made up of functionally diverse, metal-dependent proteins that contain a triosephosphate isomerase (TIM)-like barrel fold in their catalytic domains (60). They are important for amino acid and nucleotide metabolism, as well as biodegradation of industrial compounds, pesticides, and other chemicals. Although chlorhexidine treatment resulted in a 2-fold increase in amidohydrolase abundance in both WT15 and MT51 biofilms (including proteins involved in protein synthesis, folding, and stabilization, such as GroEL, ClpP protease, aspartyl/glutamyl-tRNA amidotransferase, and elongation factors Tu and Ts), chlorhexidine degradation studies using ^14^C-radiolabeled chlorhexidine demonstrated that WT15 could not mineralize chlorhexidine (31), though chlorhexidine degradation by other bacterial species is known (61).

A few proteins associated with energy production, nucleotide transport and metabolism were also found to be up-regulated (Table 3) in the presence of chlorhexidine. Their increase in abundance may be due to the higher energy demand for energy-dependent mechanisms of detoxification or adaptation to chlorhexidine. These proteins were also found to be more abundant in a *B. cenocepacia* strain that was highly resistant to different classes of antimicrobials (62).

Consistent with the observed shift in fatty acid composition, several enzymes associated with fatty acid synthesis and metabolic processes (Table 3) were affected by chlorhexidine treatment in both strains. Microorganisms often adapt to environmental stress by changing the type and composition of membrane fatty acids (63), and the induction of these enzymes in *D. acidovorans* biofilms suggests this as a contributing adaptation mechanism to chlorhexidine. For example, increased expression of malonyl-CoA:ACP transacylase indicates increased fatty acid synthesis, which means increased consumption of acetyl-CoA, as malonyl-CoA is derived from acetyl-CoA in the first step of fatty acid synthesis (64, 65). Consumption of acetyl-CoA for such purposes would decrease the production of poly-β-hydroxybutyrate (PHB) in PHB-producing organisms such as *D. acidovorans* (66), consistent with the observed decreased expression of phasin proteins, which are known to be produced only during production of PHB (67).

There is also a convincing indication of the involvement of several membrane proteins in chlorhexidine tolerance in *D. acidovorans* biofilms. A basic membrane lipoprotein (spot no. 371) (Table 3), which is an outer membrane protein, was highly up-regulated in both WT15 and MT51 chlorhexidine-treated biofilms. Bacterial lipoproteins are known to have many functions, some of which include transport, signaling, antibiotic resistance, conjugation, and protein secretion. Their involvement in microbial antibiotic resistance has been reported previously (68, 69). A possible role for quorum sensing in the response to chlorhexidine is also suggested due to the down-regulation of the two-component LuxR family transcriptional regulator, especially in WT15 biofilms treated with chlorhexidine.

The DIGE analysis indicates that overall chlorhexidine tolerance in *D. acidovorans* is an outcome of the effects of various antimicrobial resistance mechanisms at the molecular level, as has previously been reported for triclosan resistance in *Salmonella enterica* serovar Typhimurium (14). Of these, stress proteins, chaperones, and proteins involved in fatty acid metabolic processes and possibly in membrane stability appear to play a very important role in chlorhexidine tolerance in *D. acidovorans* biofilms.

## MATERIALS AND METHODS

### Bacteria, culture conditions, and MIC determination.

A chlorhexidine-tolerant strain (WT15) of *D. acidovorans* (MIC = 15 µg/ml) was isolated and characterized from South Saskatchewan River water biofilms (31). Resistance to biocides is often referred to as tolerance (31). Based on comparison of the chlorhexidine MIC for WT15 *D. acidovorans* (15 µg/ml) with MICs from other reported studies, WT15 can be considered tolerant to chlorhexidine. The chlorhexidine-susceptible mutant (MT51) (MIC = 1 µg/ml) was created from WT15 using Tn*5* transposon mutagenesis and has been characterized in detail (31). For biofilm cultivation purposes, the wild-type and mutant strains were grown from frozen stock cultures that were stored in 10% (vol/vol) glycerol at −80°C on full-strength tryptic soy agar (TSA) plates and incubated overnight at room temperature (22 ± 2°C). Well-isolated colonies were then grown in 1% TSB until mid-log phase (optical density of 0.4 at 600 nm) and subsequently used to inoculate the flow cells set up for the study.

*Delftia acidovorans* strains were screened for antibiotic resistance by using Sensititre CMV1AGNF plates (TREK Diagnostic Systems, Thermo Scientific, Waltham, MA) and the microdilution method of Andrews (70). The plates contained 17 antimicrobial agents added to 96 wells at appropriate 2-fold dilutions (i.e., 64, 32, 16, 8, 4, 2.1, 0.5, 0.25, and 0.12 µg/ml) following the National Antimicrobial Resistance Monitoring System of the Centers for Disease Control and Prevention. Each well of the microtiter plate was inoculated according to the manufacturer’s instructions, followed by room temperature incubation for 24 h. The MIC was manually determined for each isolate as the lowest concentration of each antibiotic that inhibited visible growth. MICs for chlorhexidine were determined using the same microdilution scheme. Subsequently, a finer set of chlorhexidine concentrations was used (i.e., 9, 10, 11, 12, 13, 14, and 15 µg/ml). The MIC breakpoints were determined according to Clinical and Laboratory Standards Institute (formerly the National Committee for Clinical Laboratory Standards) standards M100 (71) and M31 (72).

### Identification of the transposon insertion site.

The gene interrupted by transposon (Tn*5*) insertion in *D. acidovorans* (MT51) was identified by the rapid amplification of transposon ends (RATE) technique, as described by Karlyshev et al. (73). Amplified products were analyzed by electrophoresis on a 1% agarose gel in 1× Tris-acetate-EDTA (TAE) buffer containing 0.5 µg/ml ethidium bromide. The product band was purified using the QIAquick gel purification kit (Qiagen, Mississauga, ON, Canada) as per the manufacturer’s instructions and then sent to the National Research Council of Canada, Saskatoon, Saskatchewan, Canada, for sequencing. The sequence of the disrupted gene (*tolQ*) from WT15 was then compared with sequences published in the NCBI Nucleotide Database. TolQ has been shown to be an integral cytoplasmic membrane protein required for maintenance of the integrity of the bacterial envelope in *E. coli* (34).

### Cultivation of biofilms.

Wild-type and mutant biofilms were cultivated in multichannel flow cells, as explained by Rema et al. (31). To evaluate the effect of chlorhexidine, established (24-h) biofilms were treated for another 24-h period with sterile medium supplemented with either sub-MIC (10 µg/ml) or above-MIC (30 µg/ml) chlorhexidine concentrations. Biofilms that were not exposed to chlorhexidine and were grown under similar conditions for 48 h served as controls.

### TEM.

Biofilm cells were aseptically scraped from flow cells, and the material was transferred directly to sterile microcentrifuge tubes. The tubes were couriered on ice to the Electron Microscope Facility, McMaster University, ON, Canada, where further processing of the samples was immediately performed, as per the procedure followed by Lawrence et al. (74), with a few modifications. Ultrathin sections were cut with a diamond knife mounted on a Leica UCT Ultramicrotome and placed on TEM grids (Marivac Canada, Canton de Gore, Quebec, Canada). Lastly, sample thin sections were poststained with uranyl acetate, followed by Reynold’s lead citrate staining, and then viewed on a JEOL JEM 1200 EX TEMSCAN transmission electron microscope (JEOL, Peabody, MA).

### Cellular fatty acid analysis.

To determine whether chlorhexidine treatment influenced fatty acid composition of the cells, the following analysis was conducted. Biofilms were cultivated in the presence and absence of chlorhexidine (10 µg/ml) as described above. Biofilm cells were then scraped from the flow cell and collected in a 15-ml centrifuge tube, followed by centrifugation at 4,000 rpm for 10 min. Total fatty acids were extracted from 40 to 50 mg (wet weight) of cell pellet and methyl-esterified as described by Annous et al. (46). A Hewlett-Packard 5890 series 2 gas-liquid chromatograph (Hewlett-Packard, Avondale, PA) equipped with a flame ionization detector and a capillary column (Ultra 2; Hewlett-Packard catalog no. 19091B-102; cross-linked 5% phenyl-methyl silicone; 25 m by 0.22 mm [inside diameter]; film thickness, 0.33 mm; phase ratio, 150) with hydrogen as the carrier gas was used for separation and detection of fatty acid methyl esters (FAMEs). The FAME peaks were automatically integrated by Hewlett-Packard 3365 ChemStation software, and individual fatty acids were identified with the MIDI microbial identification software (Sherlock TSBA Library version 3.80; Microbial ID, Inc., Newark, DE).

### DIGE analysis of total cellular proteins. (i) Experimental design, sample preparation and Cy dye labeling.

Control and chlorhexidine-treated MT51 and WT15 biofilms grown for 48 h under flow conditions were aseptically recovered for extraction of protein, as previously described (75). In total, there were six biofilm treatment groups (WT15 control [designated WC], MT51 control [MC], WT15 treated at 10 µg/ml chlorhexidine [W10], WT15 treated at 30 µg/ml chlorhexidine [W30], MT51 treated at 10 µg/ml chlorhexidine [M10], and MT51 treated at 30 µg/ml chlorhexidine [M30]) with three biological replicates for each group, prepared independently from separate experiments. Sample preparation for DIGE analysis, which included cell lysis and protein extraction and solubilization, was carried out as detailed previously (39). The dye-binding assay of Bradford (76) was then performed to quantify the extracted cellular proteins by using a protein assay kit (Bio-Rad Laboratories, Hercules, CA). The extracted protein was further precipitated using the 2D cleanup kit (GE Healthcare, Mississauga, ON, Canada) as per the supplier’s protocol and resuspended in cell lysis buffer (7 M urea, 2 M thiourea, 4% wt/vol CHAPS {3-[(3-cholamidopropyl)-dimethylammonio]-1-propanesulfonate}, 30 mM Tris-Cl [pH 9.0]) to a final concentration of 5 µg/µl. The pH of the protein samples was adjusted to 8, and a total of 50 µg protein per sample was used for labeling reactions. The pooled internal standard was prepared by mixing equal amounts of protein from all treatments and dispensing them in 50-µg aliquots prior to labeling.

Protein samples were labeled using fluorescent cyanine dyes (GE Healthcare) in accordance with the manufacturer’s protocols. Cyanine dyes (Cy3 and Cy5) were freshly reconstituted in dimethylformamide and added to the labeling reaction mixtures at a ratio of 400 pmol dye to 50 µg protein. Labeling reactions were performed in the dark for 30 min and on ice, after which the reactions were terminated by the addition of 10 mM lysine (1 µl per 400 pmol dye). Each of the three replicates within a group was labeled with Cy3, Cy5, and Cy3 or Cy5. The pooled internal standard was labeled with Cy2 fluorescent dye. Sample multiplexing was also randomized to produce unbiased results.

### (ii) Two-dimensional gel electrophoresis.

For every gel, 50 µg each of Cy3-labeled and Cy5-labeled protein samples was mixed with the Cy2-labeled pooled standard, added to rehydration buffer (7 M urea, 2 M thiourea, 2% [wt/vol] CHAPS, 13 mM dithiothreitol [DTT], 0.5% immobilized pH gradient [IPG] buffer pH 4 to 7) to a total volume of 450 µl, loaded onto 24-cm, pH 4 to 7 IPG strips (GE Healthcare), and left overnight for rehydration. Isoelectric focusing (IEF) was performed using an Ettan IPGphor 3 (GE Healthcare) apparatus for a total of 57,500 V ⋅ h at 75 µA and 20°C. The focused IPG strips were equilibrated for 15 min first with freshly added DTT (0.5% wt/vol) and then with iodoacetamide (4.5% wt/vol) for another 15 min in equilibration buffer (50 mM Tris-Cl [pH 8.8], 6 M urea, 30% glycerol, 2% [wt/vol] SDS, and 0.02% bromophenol blue). Second-dimension electrophoresis was performed on 12.5% polyacrylamide gels using the Ettan DALT Six apparatus (GE Healthcare). Gels were run at 1 W per gel for 1 h and at 17 W per gel at 20°C until the bromophenol blue tracking front had just run off the bottom of the gels.

### (iii) Image acquisition and data analysis.

Cy2, Cy3, and Cy5 images for each gel were scanned at excitation and emission wavelengths of 488 and 520 nm, 532 and 580 nm, and 633 and 670 nm, respectively, at a 100-µm resolution using a Typhoon FLA9000 scanner (GE Healthcare). The images were then cropped using ImageQuant v5.0 and processed using DeCyder v7.0 differential analysis software (GE Healthcare), as per the manufacturer’s protocol. The differential in-gel analysis module of DeCyder software was used for spot codetection and quantitation. The biological variation analysis (BVA) module was used for intergel matching of internal standard and samples across all gels and allowed quantitative comparisons of protein expression across all gels. ANOVA and Student’s *t* tests were performed between different treatment groups. The extended data analysis (EDA) module of the DeCyder software was also used for multivariate analysis of protein expression data, after which principal component analysis (PCA) was performed to find experimental outliers and patterns in expression data. DIGE data were exported and compared by PCA with PRIMER v6 software. Statistical analyses of PCA scores generated from the first two component axes were run using an analysis of similarity (ANOSIM) with PRIMER v6 software (PrimerE Ltd., Lutton, United Kingdom). The following criteria was used to select proteins of interest: (i) spots that showed a significant change in expression (*P* < 0.01 and a difference of more than 1.5-fold), (ii) spots present in all gel images, and (iii) spots that gave a *q* score of more than 70 in cluster analysis. Finally, matches and data quality of proteins of interest were manually checked to avoid false positives. Forty-five spots were then marked as spots of interest to be chosen for identification using mass spectrometry.

### (iv) Protein identification.

After second-dimension electrophoresis, pick gels were prepared for picking proteins of interest by staining with Deep Purple fluorescent dye following the supplier’s protocol (GE Healthcare). The gels were scanned in a Typhoon FLA9000 scanner at a 100-µm resolution using 532 and 560 excitation and emission wavelengths, respectively. The preparative and analytical images were matched using the BVA module of the DeCyder software. Reference markers and spot-picking locations were designated and edited as required. The pick list was then exported to the Ettan spot picker (GE Healthcare), where the protein spots of interest were excised from the gel following the manufacturer’s protocol. The excised spot plugs were transferred to a 96-well microtiter plate, destained, and digested using trypsin in the MassPrep II proteomics Workstation (Micromass, United Kingdom) as per the method described by Sheoran et al. (77). The proteins were then identified by liquid chromatography nanoelectrospray ionization mass spectrometry (LC-ESI-MS). For LC-ESI-MS analysis, a quadrupole time-of-flight (Q-TOF) Global Ultima mass spectrometer (Micromass, Manchester, United Kingdom) equipped with a nanoelectrospray (ESI) source and interfaced with a nanoAcquity ultraperformance liquid chromatography (UPLC) solvent delivery system (Waters, Milford, MA) was used. The data generated from LC-ESI-MS was processed with the ProteinLynx Global Server 2.4 (Waters) and subsequently submitted to Mascot (Matrix Science Ltd., London, United Kingdom) for peptide searching against the National Center for Biotechnology Information protein database. Gene ontology (GO) enrichment analysis was conducted using the Database for Annotation, Visualization and Integrated Discovery (DAVID) to identify the biological processes and molecular functions of the proteins thus identified (78).

### Nucleotide sequence accession number.

The sequence data for *tolQ* were submitted to the GenBank database under accession number KT988307. 
